# Third Occipital Nerve Block and Cooled Radiofrequency Ablation for Managing Hemicrania Continua: A Case Report

**DOI:** 10.7759/cureus.82114

**Published:** 2025-04-11

**Authors:** Cody Barbari, Dev Patel, Jackson Cohen

**Affiliations:** 1 Physical Medicine and Rehabilitation, Memorial Healthcare System, Hollywood, USA

**Keywords:** headache, hemicrania continua, medial branch block, radiofrequency ablation, third occipital nerve

## Abstract

Hemicrania continua is a rare and debilitating headache disorder characterized by continuous, unilateral pain that responds to indomethacin but is often resistant to other treatments. This report presents the case of a 26-year-old female patient with refractory hemicrania continua and chronic migraine who achieved significant pain relief following a fluoroscopy-guided third occipital nerve block and subsequent radiofrequency ablation (RFA) of the C2-C3 facet joint. The procedure resulted in an immediate reduction of pain as evident from a reduction in the Visual Analog Scale (VAS) score from 10/10 to 0/10, with sustained relief (VAS 2/10) at three months and notable improvement in the patient's quality of life. This case highlights the potential efficacy of targeting the third occipital nerve for the management of hemicrania continua, with thermal RFA (COOLIEF, Avanos Medical, Inc., Alpharetta, GA) offering prolonged relief by ablating nociceptive fibers. Given the emerging evidence supporting the involvement of the third occipital nerve in headache pathophysiology, the third occipital nerve block and RFA represent promising strategies for refractory cases.

## Introduction

Hemicrania continua (HC), classified under trigeminal autonomic cephalalgias (TACs), is a distinct primary headache disorder, defined by persistent and strictly unilateral pain that responds to indomethacin. Although indomethacin can be an effective form of treatment for HC, it is contraindicated in patients with gastrointestinal disturbances and often leads to noncompliance with the medication [1-3]. In some cases, patients experience refractory symptoms despite extensive treatment, necessitating alternative interventional approaches. Traditional therapies include migraine analgesics, botulinum toxin injections, and nerve blocks targeting the sphenopalatine ganglia, supraorbital, and occipital nerves. Guerrero et al. (2012) showed that anesthetic blocks or corticosteroid injections may be an effective alternative for HC patients who show tenderness in the supraorbital nerve, greater occipital nerve, or trochlear area [2].

While corticosteroid injections and nerve blocks can provide a great deal of temporary relief in patients with HC, a more permanent solution, such as an RFA, is desirable to treat this debilitating condition. Chua et al. (2011) explored the mechanisms and efficacy of pulsed and conventional thermal RFA, demonstrating that thermal RFA creates a neurodestructive lesion that disrupts nociceptive pathways more effectively than pulsed RFA. Their review also emphasized the potential for thermal RFA to provide longer-lasting results in appropriately selected headache syndromes, supporting the rationale for its application in HC [4]. Usmani et al. (2018) compared the efficacy of conventional radiofrequency with thermocoagulation and pulsed radiofrequency for ganglion impar block for perineal pain in a prospective, randomized, double-blind study [5]. An intergroup comparison also showed significantly better pain relief in the thermal RFA group compared to the pulsed RFA group. At the end of follow-up, 28 patients (82%) in the conventional thermal radiofrequency (CRF) group and four patients (13%) in the pulsed radiofrequency (PRF) group had significantly improved pain, as assessed by the subjective patient satisfaction questionnaire [5]. Weyker et al. (2012) performed pulsed RFA of the supraorbital nerve with a tip temperature of 42°C for 120 s; however, the patient returned in two weeks with minimal improvement in pain. The decision was made to repeat continuous thermal RFA of the right supraorbital nerve with a maximum tip temperature of 80°C for 60 s. The patient experienced a complete resolution of symptoms after four days of this treatment with no requirement for posttreatment analgesics and the patient remained pain-free for over 12 months [6]. These studies reveal that thermal RFA yielded superior results over pulsed RFA. It supports the authors’ use of thermal RFA method to achieve therapeutic results in a patient with HC.

## Case presentation

A 26-year-old female patient presented with chronic, right-sided headache pain that began at the age of eight following a mononucleosis-like illness confirmed by elevated Epstein-Barr virus (EBV) titers. Initially, her headaches were unilateral and associated with photophobia, phonophobia, altered smell, movement sensitivity, nausea, lightheadedness, and difficulty concentrating. By age 11, she developed severe, stabbing pain in the right eye accompanied by excessive lacrimation, nasal congestion, and ptosis. She was initially diagnosed with cluster headaches due to her responsiveness to oxygen within 5 min and lack of response to indomethacin. 

**Figure 1:**
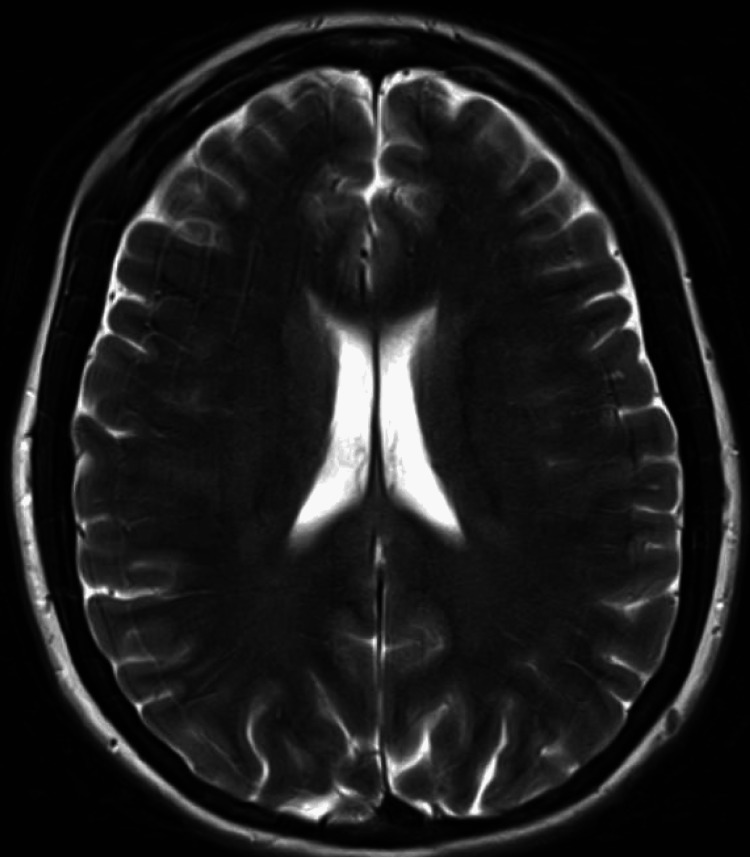
Axial T2-Weighted Sequence MRI of the Brain Axial T2-weighted MRI of the brain showing no acute pathology, with no significant changes compared to prior imaging.

**Figure 2:**
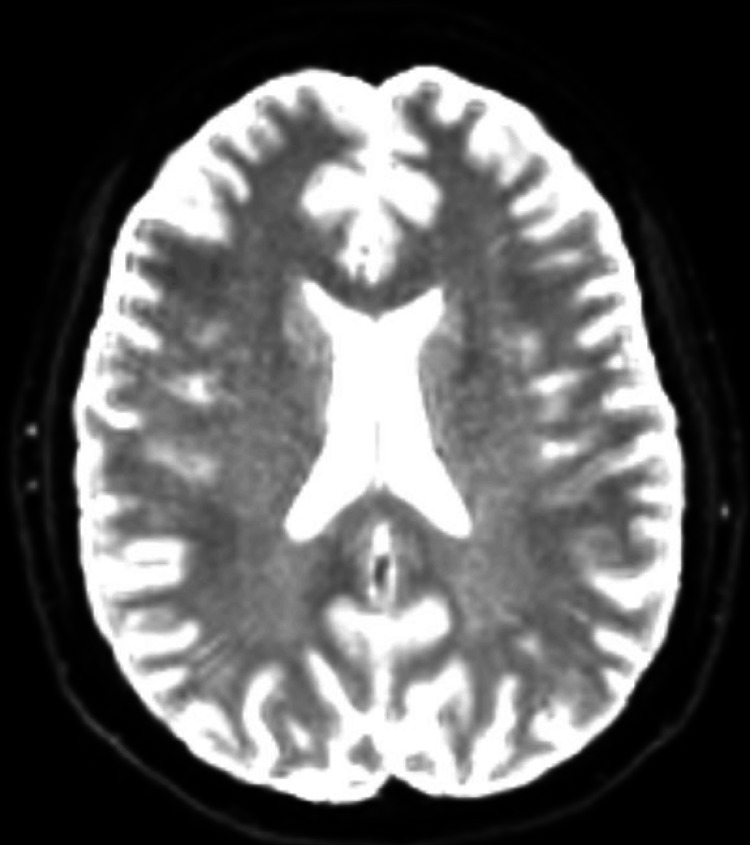
Diffusion-Weighted Imaging (DWI) Sequence MRI of the Brain Diffusion-weighted imaging MRI of the brain showing no evidence of acute pathology, including no restricted diffusion to suggest acute infarction.

Years later, a retrial of indomethacin yielded complete symptom relief, leading to a revised diagnosis of HC. This diagnosis was confirmed based on the International Classification of Headache Disorders (ICHD-3b) criteria: unilateral, continuous daily headache for over three months with moderate to severe intensity, associated autonomic features, and a complete response to indomethacin [7]. Despite this, the patient experienced treatment failure after multiple unsuccessful attempts at weaning off indomethacin. Recent MRI brain imaging showed no acute pathology compared to prior scans (Figures 1, 2). Over the following decade, her headaches progressively worsened in duration and frequency, necessitating ongoing evaluation and management due to persistent refractory symptoms.

Along the patient's journey with numerous specialists over the years, she had failed numerous conservative and interventional treatments including various migraine analgesics, botulinum toxin injections, indomethacin, sphenopalatine ganglion blocks, supraorbital and occipital nerve stimulators, and vagal nerve stimulation. Despite these therapies, her Visual Analog Scale (VAS) score remained at a 10/10 prior to intervention, and the headaches significantly impacted her quality of life.

After consultation, the decision was made to perform a third occipital nerve block under fluoroscopy targeting the right cervical medial branch nerve at the C2-C3 facet joint, followed by RFA. This approach was based on emerging evidence suggesting that third occipital nerve involvement could contribute to HC symptomatology in patients unresponsive to other therapies.

**Figure 3:**
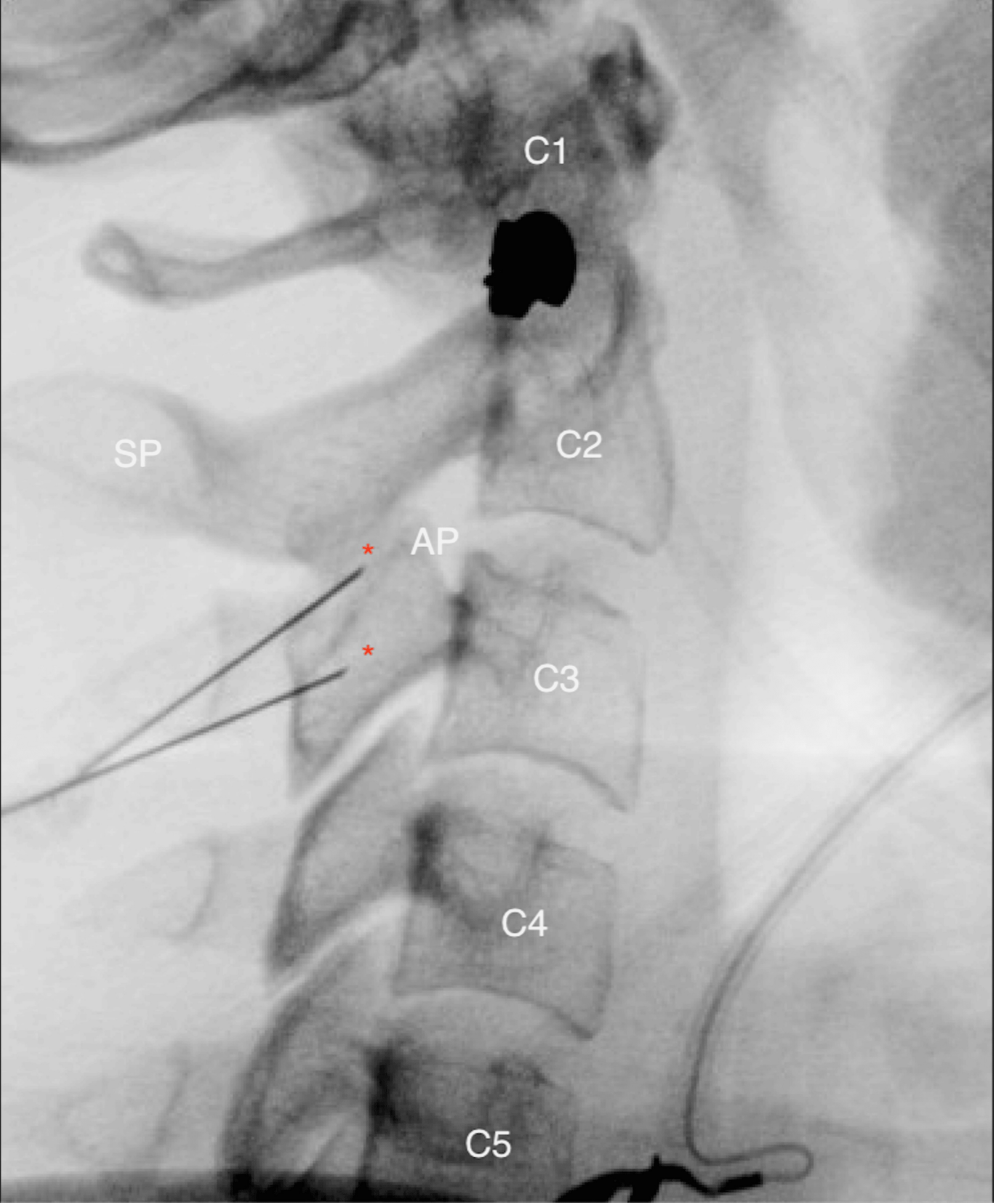
True lateral view of the cervical spine to identify the articular pillar at C3 and the C2-C3 neural foramen opposite base. Target sites are marked with an asterisk. Articular process (AP), spinous process (SP)

Following two successful diagnostic nerve blocks that provided over 80% pain relief (Figure 3), the patient was scheduled to undergo RFA (COOLIEF, Avanos Medical, Inc., Alpharetta, GA). The details of the RFA procedure are described below. 

The patient was placed in the left lateral decubitus position, and surface anatomy was identified. The skin over the planned injection site was prepared with a sterile technique and allowed to dry. The area was draped in a sterile fashion, and the correct patient, procedure, side, and site were confirmed per the institutional protocol.

**Figure 4:**
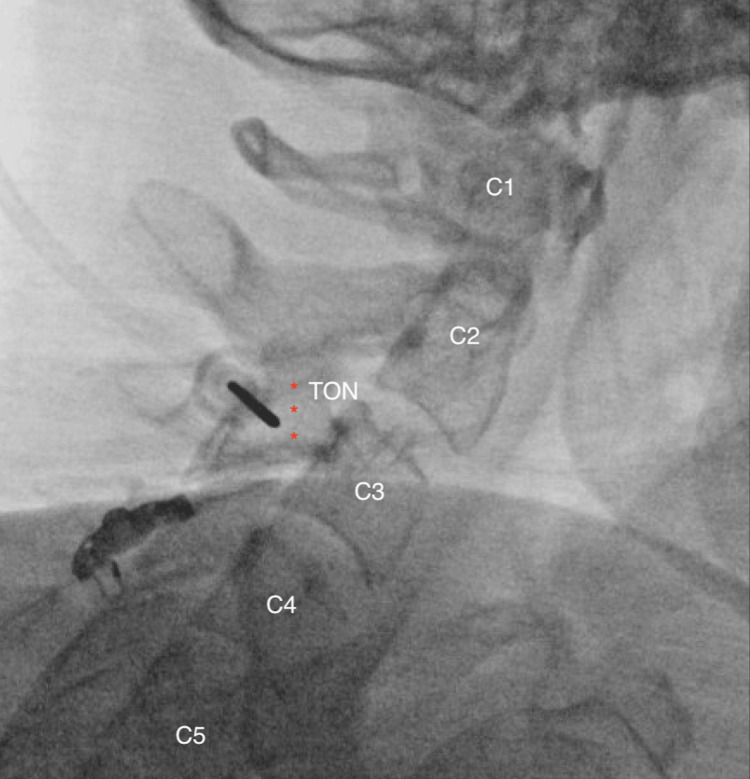
Lateral view of the cervical spine illustrating the RFA needle tip positioned at the lower target point for a third occipital nerve injection. Asterisks indicate the hypothetical course of the TON and the intended ablation site. Third occipital nerve (TON)

Using fluoroscopic guidance, a true lateral view of the right articular pillars at the C2 and C3 vertebrae was obtained (Figure 4). The skin was infiltrated at the entry point with 1% lidocaine via a 25-gauge, 1½-inch needle to provide local anesthesia. A 17-gauge RFA needle with a 2 mm active tip was advanced to the center of the rhomboid structure at the C3 and the C2-C3 junction under fluoroscopic guidance until bone contact was achieved. The lateral view confirmed that the needle placement was distant from the intervertebral foramen, ensuring safety (Figure 4).

The RF cannula was introduced through the RFA needle, and motor testing was performed to confirm proper placement, with no motor response detected up to 2 V. Following this, the RF cannula was removed, and after negative aspiration, 1.5 ml of 2% lidocaine was injected to further anesthetize the site. The RFA cannula was then reinserted, and RFA was performed at 60°C for 150 s, with a maximum tip temperature of 80°C.

Following the RFA, and after negative aspiration, a mixture of 0.5 ml dexamethasone (10 mg/ml) and 0.5 ml of 1% lidocaine was injected through the RFA needle. No evidence of intravascular or intrathecal injection was observed. The needles were removed intact, and the patient’s skin was cleansed and dried before placing a bandage over the operative site. 

Immediately following the procedure, the patient's VAS score decreased to 0/10. At a two-week and three-month follow-up, the patient reported sustained pain relief, with a VAS score of 2/10 and significant improvement in quality of life. She reported improved sleep and reduced headache frequency, indicating a positive response to the intervention.

## Discussion

HC is a challenging condition to treat due to its unrelenting nature and often refractory response to standard treatments. Traditional management options for HC include indomethacin, which is typically effective but not well-tolerated in all patients. Interventional approaches, such as sphenopalatine ganglion (SPG) blocks, occipital nerve blocks, and nerve stimulators, have shown mixed results. The third occipital nerve has been implicated in certain headache syndromes due to its anatomical proximity to cervical and cranial nerve pathways involved in headache pathophysiology.

This case highlights the potential efficacy of targeting the third occipital nerve through both nerve blocks and RFA. The sustained reduction in pain experienced by the patient in this case study suggests that this approach may provide longer-lasting relief for individuals with refractory HC. RFA, which uses thermal energy to ablate the nerve fibers, may offer extended pain relief by disrupting pain signal transmission along the third occipital nerve. RFA may also offer advantages over nerve stimulation techniques in terms of invasiveness, cost, and duration of pain relief [6,8].

Emerging evidence supports the use of cervical medial branch blocks and RFA in managing chronic headache disorders, but further research is necessary to establish standardized protocols and determine optimal patient selection criteria. Miller et al. (2016) found that 34.5% of patients with HC responded to occipital nerve blocks, suggesting occipital nerve involvement in some cases [8]. Androulakis et al. (2016) successfully treated a 52-year-old woman with a seven-year history of HC, who was unable to tolerate indomethacin, using a local bupivacaine SPG block. However, it is important to note that this treatment required repeated visits and blocks, administered twice a week for six weeks, which may not be the most efficient approach [9]. Additionally, Hamer et al. (2016) reported successful treatment of recurrent cervicogenic headaches with C2 dorsal root ganglion and third occipital nerve RFA in 23 cases, further supporting the approach used in this study [10]. Granato et al. (2018) demonstrated the potential of occipital nerve interventions in a prospective study of 20 patients with HC treated with occipital nerve stimulation, finding that 85% of the patients had >50% pain reduction at 38.5 months follow-up [11]. While not specific to HC, Choi et al. (2015) showed significant pain reduction lasting six months in patients with occipital neuralgia treated with pulsed radiofrequency of the greater and lesser occipital nerves [12]. 

The technique of placing the patient prone with lateral head positioning adopted in this study offered a safe and effective approach for accessing the medial branches while minimizing potential complications. This positioning allowed for precise anatomical targeting and reduced the risk of inadvertent injury to surrounding structures. Compared to other studies, such as those by Androulakis et al., Hamer and Purath, and Granato et al., which utilized varied patient positions like lateral decubitus or supine, the approach adopted by the authors in this study provided superior stabilization and visibility of the medial branch targets. In these studies, reliance on alternative positioning may have introduced variability in needle placement and lesion accuracy.

Although thermal RFA has been more commonly employed in previous studies, the authors opted for cooled RFA due to its ability to create larger and more spherical lesions, which may increase the likelihood of effectively capturing the anatomically variable course of the third occipital nerve [13]. Cooled RFA allows for internal electrode cooling, which reduces peak tissue temperatures and minimizes charring, thereby producing more consistent lesion shapes and volumes, the factors particularly important when targeting a small, anatomically complex sensory branch such as the third occipital nerve [14]. This approach aligns with the authors' goal of providing longer-lasting pain relief and improved function in patients with refractory cervicogenic headache and while more comparative research is needed, this case demonstrates the potential clinical utility of cooled RFA in this setting.

## Conclusions

The targeted third occipital nerve block with subsequent RFA provided significant, durable pain relief for a patient with refractory HC. This interventional approach may serve as an alternative for patients unresponsive to conventional treatments. Continued monitoring and follow-up are essential to assess the long-term efficacy of this treatment modality. Further studies are needed to validate this approach and expand its applicability to a broader patient population, building upon the results seen in recent literature.

## References

[1] Pascual J (2009). Treatment of hemicrania continua by occipital nerve stimulation with a bion device. Curr Pain Headache Rep.

[2] Guerrero ÁL, Herrero-Velázquez S, Peñas ML, Mulero P, Pedraza MI, Cortijo E, Fernández R (2012). Peripheral nerve blocks: a therapeutic alternative for hemicrania continua. Cephalalgia.

[3] Cittadini E, Goadsby PJ (2010). Hemicrania continua: a clinical study of 39 patients with diagnostic implications. Brain.

[4] Chua NH, Vissers KC, Sluijter ME (2011). Pulsed radiofrequency treatment in interventional pain management: mechanisms and potential indications - a review. Acta Neurochir (Wien).

[5] Usmani H, Dureja GP, Andleeb R, Tauheed N, Asif N (2018). Conventional radiofrequency thermocoagulation vs pulsed radiofrequency neuromodulation of ganglion impar in chronic perineal pain of nononcological origin. Pain Med.

[6] Weyker P, Webb C, Mathew L (2012). Radiofrequency ablation of the supraorbital nerve in the treatment algorithm of hemicrania continua. Pain Physician.

[7] Prakash S, Patel P (2017). Hemicrania continua: clinical review, diagnosis and management. J Pain Res.

[8] Miller S, Watkins L, Matharu MS (2017). Treatment of intractable hemicrania continua by occipital nerve stimulation. J Neurol Neurosurg Psychiatry.

[9] Androulakis XM, Krebs KA, Ashkenazi A (2016). Hemicrania continua may respond to repetitive sphenopalatine ganglion block: a case report. Headache.

[10] Hamer JF, Purath TA (2016). Repeat RF ablation of C2 and third occipital nerves for recurrent occipital neuralgia and cervicogenic headaches. World J Neurosci.

[11] Govindappagari S, Grossman TB, Dayal AK, Grosberg BM, Vollbracht S, Robbins MS (2014). Peripheral nerve blocks in the treatment of migraine in pregnancy. Obstet Gynecol.

[12] Choi I, Jeon SR (2016). Neuralgias of the head: occipital neuralgia. J Korean Med Sci.

[13] MacVicar J, Borowczyk JM, MacVicar AM, Loughnan BM, Bogduk N (2012). Cervical medial branch radiofrequency neurotomy in New Zealand. Pain Med.

[14] McCormick ZL, Choi H, Reddy R (2019). Randomized prospective trial of cooled versus traditional radiofrequency ablation of the medial branch nerves for the treatment of lumbar facet joint pain. Reg Anesth Pain Med.

